# Mortality in Brazilian federal highway police officers: time series from 2001 to 2020

**DOI:** 10.11606/s1518-8787.2022056004210

**Published:** 2022-09-12

**Authors:** Eduardo Frio Marins, Rodrigo Wiltgen Ferreira, Flávio Castagna de Freitas, Geovana Ferreira de Andrade Alves Dutra, José Rossy e Vasconcelos, Eduardo Lucia Caputo

**Affiliations:** I Polícia Rodoviária Federal Brasília DF Brasil Polícia Rodoviária Federal. Brasília, DF, Brasil; II Universidade Federal de Pelotas Escola Superior de Educação Física Programa de Pós-Graduação em Educação Física Pelotas RS Brasil Universidade Federal de Pelotas. Escola Superior de Educação Física. Programa de Pós-Graduação em Educação Física. Pelotas, RS, Brasil; III Instituto Federal de Educação, Ciência e Tecnologia Farroupilha São Borja RS Brasil Instituto Federal de Educação, Ciência e Tecnologia Farroupilha. Campus São Borja. São Borja, RS, Brasil

**Keywords:** Police, Mortality, trends, Cause of Death, Time Series Studies

## Abstract

**OBJECTIVE:**

To analyze the mortality trend from all causes in Brazilian federal highway police officers from 2001 to 2020.

**METHODS:**

This is an ecological time-series study based on mortality official data from the Brazilian federal highway police registry system and death certificates from the federal registry system. Deaths of active police officers from 2001 to 2020 were assessed. We performed a descriptive analysis reporting proportions and incidence rates per 1,000 police officers. The chi-square test was used for bivariate analyzes and Prais-Winsten regression was used for trend analysis.

**RESULTS:**

Among 346 deaths, 146 were from natural and 189 from unnatural causes (11 were from undefined causes). Most deaths occurred among police officers who were men (n = 333; 96.3%), over 35 years old (n = 265; 76.6%), whose service time was up to 15 years (n = 185; 53.5%), living in Northeast Brazil, and from unnatural causes (n = 189; 56.4%). The absolute number of deaths presented a decreasing trend throughout the series (p = -0.78; 95%CI: -1.03 to -0.5). Traffic accidents (n = 96; 28.7%), cardiovascular diseases (n = 58; 17.3%), interpersonal violence (n = 51; 15.2%), suicides (n = 35; 10.5%), and malignant neoplasms (n = 35; 10.4%) were the main causes of death. Most natural deaths occurred among police officers who were 51–73 years old (68.3%; 95%CI: 58.6 to 76.7) and worked more than 26 years (64.7%; 95%CI: 52.7 to 75.1), while most unnatural deaths occurred among officers who were 19–35 years old (87.3%; 95%CI: 78.0 to 93.1) and worked up to 15 years (70.2%; 95%CI: 63.1 to 76.4).

**CONCLUSION:**

The mortality trend in Brazilian federal highway police officers decreased within the period studied. Understanding mortality causes may help to develop policies for disease prevention and health protection of police officers.

## INTRODUCTION

During their work routine, police officers perform a wide range of tasks that are risky and potentially harmful to health^1 , 2^ . Intense physical efforts (e.g., pursuing criminals who resists arrest), traumatic events (e.g., attending accidents with victims), sound and chemical stress (e.g., exposure to high levels of noise and carbon dioxide), and threats to physical and psychological health (e.g., participation in armed confrontations) are some risks inherent in this profession^1 , 3^ . Due to the characteristics of their work regime (shift work), police officers may undergo changes in circadian rhythm and sleep disorders^4 , 5^ .

A study performed with New York police officers presented a significant increase in mortality rates from all causes of death, malignant and benign neoplasms, liver cirrhosis, and all diseases of the circulatory system from 1950 to 2005^6^ . In Brazil, a study performed with Rio de Janeiro police officers (civil and military) and municipal guards showed that aggression and traffic accidents were the main causes of death from 1994 to 2004^7^ . Moreover, these workers also suffer from other health problems, such as cancer and cardiovascular diseases^2^ , and are highly exposed to risk situations inherent with their profession, which can increase the number of deaths from external causes, such as interpersonal violence and suicide^7^ .

The existence of multiple risk factors in this profession provides a compelling reason to study the main causes of mortality among police officers. However, to date literature lacks on this topic regarding police officers, especially within Brazilian police forces, such as the federal highway police (FHP)^6^ . Developing scientific knowledge to map and categorize the main causes of death in FHP officers may contribute in a great extent to decision makers when promoting public health policies, better professional training actions, and disease prevention projects, as well as in the police officers’ health promotion.

Therefore, this study aimed to describe and analyze the time series of mortality in active FHP officers from January 1, 2001 to December 31, 2020.

## METHODS

This is an observational study with an analytical and exploratory ecological design of FHP officers’ mortality time series from all over Brazil from 2001 to 2020.

Police officers who were active from January 1, 2001 to December 31, 2020, whose deaths were reported, were included in this study. Administrative staff and retired police officers were excluded.

**Figure 1:**
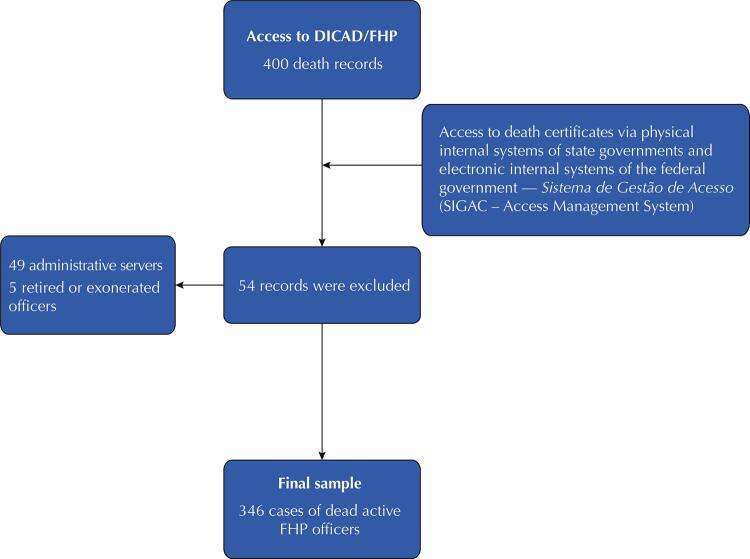
Flowchart of the sampling process to obtain data on deaths within the organizational structure of the FHP. FHP: Brazilian federal highway police; DICAD: Registration Division.

Data on deaths were assessed from the responsible departments, according to the FHP organizational structure. Figure 1 shows the sampling process.

The underlying causes of death were assessed from individuals death certificate, afterwards the authors of this study, who are health experts, (EFM, FCF, and JRVJ) grouped and coded each certificate according to the cause-of-death category and definition under the International Classification of Diseases 10^th^ Revision (ICD-10)^12^ . When the death was classified as undetermined in the death certificate, we classified as ill-defined/undetermined causes. Deaths were categorized as natural and unnatural, according to the Brazilian Ministry of Health classification.

Sex (men and women), age at the time of death (years), service time (full years), and the region of Brazil where the officer worked were the independent variables. Age and service time were later categorized based on the population distribution in each variable.

Data analyzes was based on descriptive statistics by the number of absolute and relative cases and their respective 95% confidence intervals (95%CI). Prais-Winsten regression with robust variance was used to analyze the mortality trend^13^ . Deaths absolute frequency was the outcome variable used in the model. The years of the historical series was the exposure variable. The series increased when the model coefficient was positive, decreased when it was negative, and remained unchanged when its value and zero presented no significant difference (p > 0.05)^13^ . Bivariate analyzes between the outcome and exposures of interest were performed using the chi-square test. Finally, the outcome was assessed based on its relative incidence rate, according to the number of police officers in each year studied. The 5% significance level was considered. All analyzes were performed in the statistical software Stata 15.1.

This study was approved by the Research Ethics Committee of the *Escola Superior de Educação Física* of the *Universidade Federal de Pelotas* , RS, under protocol no. 47809721.8.0000.5313.

## RESULTS

From 2001 to 2020, 346 active FHP officers died. Among these, 11 deaths (3.2%) were undetermined and were excluded from the bivariate analyzes, totaling 335 deaths with confirmed cause. The police officers included had a mean age of 44.7 years old (SD = 10.8) and worked in the FHP for 15.9 years (SD = 10.6). Most deaths during this period occurred among officers who were men, older than 35 years, worked up to 15 years, and lived in Northeast Brazil ( Table 1 ). The proportion of unnatural deaths was higher (56.4%) ( Table 1 ).

**Table 1:** Number and percentage of deaths in federal highway police officers according to sex, age, service time, region of Brazil, and cause of death, under the categorization of the Brazilian Ministry of Health. Brazil, 2001–2021 (n = 346).

	Deaths
	
	n (%)
Sex	
Men	333 (96.3)
Women	13 (3.7)
Age (years old)	
19–35	81 (23.4)
36–50	160 (46.2)
51–73	105 (30.4)
Service time (years)	
≤ 15	185 (53.5)
16–25	90 (26.0)
≥ 26	71 (20.5)
Region of Brazil	
North	35 (10.1)
Midwest	44 (12.7)
Northeast	114 (33.0)
Southeast	98 (28.3)
South	55 (15.9)
Cause of death^a^	
Natural death/disease	146 (43.6)
Unnatural death/external cause	189 (56.4)

^a^ 11 death reports were lost (undetermined cause).

**Figure 2:**
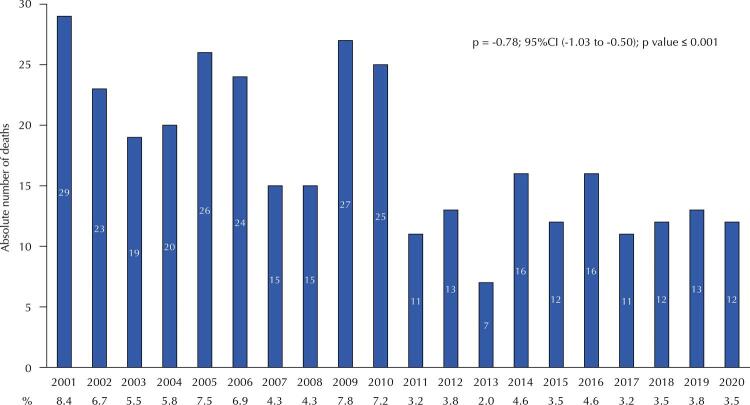
Absolute and relative frequencies (%) and Prais-Winsten regression of estimated deaths in federal highway police officers in the last 20 years (2001–2020) (n = 346).

The average number of deaths over the period was 17.4 deaths/year, ranging from seven to 29 deaths in 2013 and 2001, respectively ( Figure 2 ). The number of deaths, regarding the absolute amount and the mean value, decreased in the second decade of the period studied (2011–2020) when compared with the first (2001–2010) (12.3 and 22.3 mean deaths, respectively). Based on the Prais-Winsten regression results, we observed a significant decrease trend in the number of deaths over the period (-0.78 deaths/year) (95%CI: -1.03 to -0.50; p < 0.001).

Table 2 presents the absolute and relative values from specific causes of death, according to the Brazilian Ministry of Health classification. Traffic accidents (28.7%) were the main cause of death, followed by cardiovascular diseases (17.3%), interpersonal violence (15.2%), suicides (10.5%), and malignant neoplasms (10.4%). During the period studied, 54.4% of total deaths were from traffic accidents, interpersonal violence, and suicides (unnatural deaths), and they represent 96.3% of all unnatural deaths over the period.

**Table 2:** Characteristics of deaths in federal highway police officers in descending order from 2001 to 2020, total and stratified, according to the categorization of the Brazilian Ministry of Health (n = 335).

Characteristics	Deaths		

	Total	Natural death/disease	Unnatural death/external cause
		
	n (%)	% (95%Cl)	% (95%Cl)
Traffic accident	96 (28.7)		50.5 (43.4–57.6)
Cardiovascular diseases	58 (17.3)	39.7 (32.1–47.9)	
Interpersonal violence	51 (15.2)		26.8 (21.0–33.6)
Suicide	36 (10.5)		18.9 (14.0–25.2)
Malignant neoplasms	35 (10.4)	24.0 (17.7–31.6)	
Digestive diseases	20 (6.0)	13.7 (9.0–20.3)	
Infectious and parasitic diseases	14 (4.2)	9.6 (5.8–15.6)	
Respiratory infection	12 (3.6)	8.2 (4.7–14.0)	
Unintentional injuries	7 (2.1)		3.7 (1.8–7.6)
Respiratory diseases	4 (1.2)	2.7 (1.0–7.1)	
Endocrine diseases	3 (0.9)	2.1 (0.7–6.2)	

Table 3 shows that the proportion of deaths from unnatural causes was higher among police officers from 19 to 35 years old (87.3%; 95%CI: 78.0 to 93.1) and whose service time was up to 15 years (70.2%; 95%CI: 63.1 to 76.4). The proportion of deaths from natural causes was higher among officers from 51 to 73 years old (68.3%; 95%CI: 58.6 to 76.7) and with 26 or more years of service (64.7%; 95%CI: 52.7 to 75.1).

**Table 3:** Percentage of deaths in federal highway police officers by death category, according to age, service time, and region of Brazil. Brazil, 2001–2021 (n = 335).

	Natural death/disease	Unnatural death/external cause	p
		
	% (95%Cl)	% (95%Cl)	
Age (years old)			< 0.001
19–35	12.7 (6.9–22.0)	87.3 (78.0–93.1)^a^	
35–50	43.0 (35.4–50.9)	57.0 (49.1–64.6)	
51–73	68.3 (58.6–76.7)^a^	31.7 (23.3–41.4)	
Service time (years)			< 0.001
≤ 15	29.8 (23.6–36.9)	70.2 (63.1–76.4)^a^	
16–25	55.8 (45.1–65.9)	44.2 (34.1–54.8)	
≥ 26	64.7 (52.7–75.1)^a^	35.3 (24.9–47.3)	
Region of Brazil			0.64
North	38.7 (23.4–56.6)	61.3 (43.4–76.6)	
Midwest	36.4 (23.5–51.4)	63.6 (48.6–76.4)	
Northeast	44.6 (35.7–53.9)	55.4 (46.0–64.3)	
Southeast	48.9 (39.0–59.0)	51.1 (41.0–61.0)	
South	40.7 (28.5–54.2)	59.3 (45.8–71.5)	

95%CI: 95% confidence interval.

^a^ Statistical difference in percentage of unnatural death/external cause in relation to natural death/disease.

**Figure 3:**
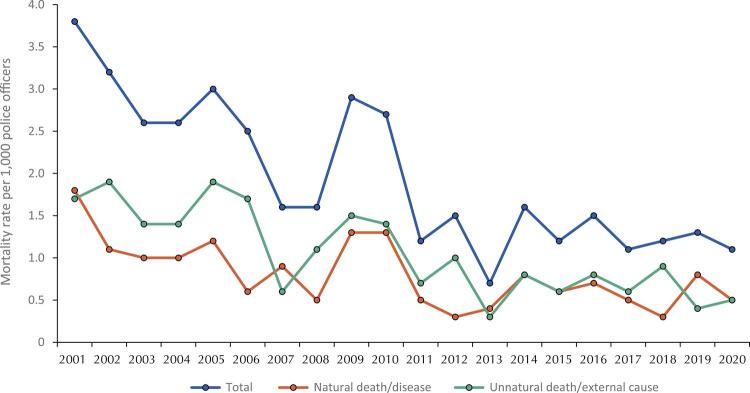
Mortality incidence rate in federal highway police officers from 2001 to 2020, total and stratified, according to the categorization of the Ministry of Health.

Figure 3 shows mortality rates from 2001 to 2020. The overall mortality rate per 1,000 police officers decreased over the period: in 2001 it was close to four deaths per 1,000 police officers; however, in 2014 was close to 1.5 deaths per 1,000 police officers. Moreover, the rate of unnatural deaths was higher in almost every year, except for 2001, 2007, 2013, and 2019, when the rate of natural deaths was higher.

## DISCUSSION

This study showed that mortality from all causes was higher among police officers who were men, over 35 years old, working for 15 years or less, and living in Northeast Brazil. Unnatural deaths were more prevalent and frequent among officers who were younger and worked for less time. Over the years, there was a decreasing trend in the number of deaths and the mortality rate decreased. Traffic accidents, cardiovascular diseases, interpersonal violence, suicides, and malignant neoplasms were the main causes of death among FHP officers.

In our study, police officers presented age and causes of death similar to those found in other forces^11 , 14^ . Merino^15^ performed an ecological time-series study from 2002 to 2006 and showed that 77% of São Paulo military police officers died from unnatural causes. Thus, police officers are exposed to risk factors specific to their profession (e.g., armed conflicts and vehicle pursuits), which may be related to this higher proportion of unnatural deaths.

Moreover, this higher percentage of unnatural deaths among officers with shorter service time corroborates the results from a study with Rio de Janeiro military police officers, in which those with shorter service time were 2.4 times more likely to die when compared to officers with longer service time^16^ . The lack of perception and experience of the risks of work activities in younger police officers may explain this relation. In novice police officers, the adrenaline produced by the unusual and their motivation for action decreases their risk perception in pursuit or confrontation moments, putting these individuals at increased risk^17^ .

As well as other police forces, FHP officers are more vulnerable to unnatural deaths (interpersonal violence, traffic accidents, and suicide) since proactive policing and prosecution of all types of crimes^18^ in federal highways are some of their competencies and they expose their physical integrity to a greater risk. Thus, understanding the police officers victimization profile allows to identify the most reliable monitoring pattern, which helps in the assessment of investments priorities in health policies for FHP officers, aiming to implement more effective actions/programs to reduce mortality and costs for public administration in general.

The FHP does not have positions related to health professionals or sectors, such as walk-in clinics, outpatient clinics, and hospitals in its official staff. However, its organizational structure includes health management sectors that act at the basic level of care, providing generic interventions (e.g., physical tests^19^ and lectures with guidelines on healthy lifestyles^20^ ), procedural interventions (e.g., the registration of medical certificates and referral to official expert examination), and crisis management/critical incident interventions (e.g., telecare for servers involved in armed conflicts and traffic accidents, and affected by covid-19).

The absolute number of deaths decreased from 2011 to 2020, which may be related to health prevention and promotion programs implementation , as well as actions in the FHP, such as: i) the *Patrulha da Saúde* (Health’s Patrol Program), which aims to promote knowledge about disease prevention measures and healthy lifestyles, according to previously published data^20^ ; ii) the inclusion of a course regarding health in the curriculum of police training courses, aiming the adoption of habits promoting the integral health of officers in training, focusing on techniques that involve the regular practice of physical exercises, healthy eating, quality of sleep, and issues related to psycho-emotional health; iii) the application of a physical fitness test, aiming to assess the physical fitness levels of police officers promoting career advancement and defining selection criteria for specific activities^21^ ; and iv) the institutional physical education program, which encourages the physical activity practice outside the work environment^19^ . Those actions will contribute to lifestyle changes and, consequently, reducing morbidity and mortality from natural causes among police officers; however, our study design did not allow us to analyze the causality between health initiatives in the FHP and mortality over the period^13^ .

Traffic accidents were the main cause of death among police officers from 2001 to 2020 (28.7%), corroborating a recent study that assessed mortality among Rio Grande do Sul military police officers from 2006 to 2016^14^ . Limeira and Donato^14^ showed that traffic accidents were the main cause of death among active police officers (41.3% of all deaths during the period studied), being ahead of deaths from interpersonal violence (homicide) and suicides, as occurred among police officers over the 20 years. Both FHP officers and military police officers are part of the proactive Brazil and its states police, respectively, and their policing activities require the constant use of cars and motorcycles (police vehicles) and permanence in places with a large volume of vehicles to maintain road safety, which can increase the risk of hit-and-run accidents. Thus, institutionalizing prevention policies, based on police officers training and research on this topic, is important to improve their behavior in traffic accidents.

Cardiovascular diseases were the main cause of death among natural deaths in the time series (39.7%), which is similar to the results of studies with New York^6^ and São Paulo^15^ police officers. Vena et al.^6^ presented a high number of deaths from diseases of the circulatory system (44.9% of all deaths) among New York police officers from 1950 to 2005. This high percentage is probably due to the high prevalence of some cardiovascular risk factors among police officers, such as long shifts, sleep disorders, psychological stress, unforeseen physical stress, and noise exposure^2^ .

Moreover, Vena et al.^9^ showed that mortality rates from arteriosclerotic heart disease and different types of cancers among U.S. police officers increased from 1950 to 1970. Similarly, Forastiere et al.^8^ , when studying a historical cohort of police officers in Rome, Italy, showed that mortality rates from ischemic heart disease, as well as some types of cancers, increased in police officers under 50 years old. Violanti et al.^10^ studied a U.S. police officers’ cohort from the 1950s to 1990s and mortality from all malignant neoplasms, as well as arteriosclerotic heart disease, significantly increased in police officers with longer service time. Therefore, these results, as well as those from our study, reinforce the relationship between age and service time and mortality from natural causes in police officers, especially from cardiovascular diseases and malignant neoplasms, which are possibly related to occupational risk factors and police officers lifestyle^2^ .

During the 20 years studied, FHP officers suicides were among the four main causes of death and was the third main cause of unnatural deaths, in accordance with previous studies with military police officers^14 , 15^ . Police officers are exposed to aspects (e.g., easy access to firearms) and specific professional situations (armed conflicts, violence, interaction with death scenes, etc.), which can be crucial to increase the risk of suicide among them^22^ . Thus, all police forces shall monitor and analyze data on suicide (its profile and death rate) to guide the development, implementation, and evaluation of public policies on police officers mental health.

Considering the trend analysis on absolute deaths number and the mortality incidence rate found in our study, the time series reduction is different when compared to the results of a study with Rio de Janeiro military police officers^16^ . Minayo et al.^16^ collected mortality rate data in these officers and showed an increasing trend from 1994 to 2004 and values ranged from 12.1 to 17 deaths per 1,000 police officers from 2000 to 2004. These values are higher when compared to the same years in our study, which is probably due to the different characteristics of FHP officers and Rio de Janeiro military police officers, since the percentage of military police officers working in another activity on their day off (mainly in private security services) is very high^16^ .

Our study has limitations. First, the quality of secondary data depend on their origin information system. In our study, we obtained death information from death certificates completed in registry offices from declarations of death filled by medical doctors. Thus, both death certificates filling and declarations of death transcription presented limitations of standardization that hindered the classification of death causes^25^ . Secondly, we lacked FHP officers’ data such as the presence of preexisting diseases, physical fitness levels, police or military activity before joining the FHP, among other information that could help in to analyzed possible risk factors for mortality. However, our study stands out for its novelty regarding the topic of mortality in police officers in Brazil, as well as the time range studied (20 years) and the use of a current period in the time series evaluation (2001–2020).

The data presented in our study allows to make the situational diagnosis of deaths in Brazilian FHP officers. Such information is important to support the development of public health policies, as well as evidence-based practical actions of professional training in the FHP.

This study shows that public health policies implemented by the FHP probably contributed to decrease the number of deaths from all causes in active police officers from 2001 to 2020. However, actions and programs related to the prevention of the main causes of death among police officers, mainly traffic accidents, cardiovascular diseases, interpersonal violence, suicides, and malignant neoplasms, shall be implemented, aiming to reach the greatest number of police officers and reduce impacts on both health and its costs.
