# Supplementary material for: Mortality in Brazilian federal highway police officers: time series from 2001 to 2020
Source: Rev Saude Publica. 2022 Sep 12;56:82. doi: 10.11606/s1518-8787.2022056004210 (PMC9469873; doi:10.11606/s1518-8787.2022056004210)
Supplement: Translation

# Translation: Mortalidade em agentes da polícia rodoviária federal: série temporal entre 2001 e 2020

**Authors:** Eduardo Frio Marins, Rodrigo Wiltgen Ferreira, Flávio Castagna de Freitas, Geovana Ferreira de Andrade Alves Dutra, José Rossy e Vasconcelos, Eduardo Lucia Caputo

**Affiliations:** I Brasil Polícia Rodoviária Federal. Brasília, DF, Brasil; II Brasil Universidade Federal de Pelotas. Escola Superior de Educação Física. Programa de Pós-Graduação em Educação Física. Pelotas, RS, Brasil; III Brasil Instituto Federal de Educação, Ciência e Tecnologia Farroupilha. Campus São Borja. São Borja, RS, Brasil

**Keywords:** Polícia, Mortalidade, tendências, Causas de Morte, Estudos de Séries Temporais

## Abstract

**OBJETIVO:**

Descrever e analisar a tendência de mortalidade, por todas as causas, em agentes da polícia rodoviária federal, entre os anos de 2001 e 2020.

**MÉTODOS:**

Trata-se de um estudo ecológico de séries temporais, baseado em dados oficiais sobre mortalidade registrados no sistema de cadastro nacional da polícia rodoviária federal e de certidões de óbitos do sistema de cadastro federal. Foram coletados os óbitos de agentes que estavam em efetivo exercício entre 2001 e 2020. Realizou-se análise descritiva, calcularam-se proporções e taxas de incidência por 1.000 policiais. Utilizou-se qui-quadrado para análises bivariadas e regressão de *Prais-Winsten* para análise de tendência.

**RESULTADOS:**

Ocorreram 346 óbitos (11 por causas indeterminadas), dos quais 146 foram por mortes naturais e 189 não naturais. A maioria das mortes ocorreu em policiais do sexo masculino (n = 333; 96,3%), acima de 35 anos (n = 265; 76,6%), tempo de serviço até 15 anos (n = 185; 53,5%), da região Nordeste e por causas não naturais (n = 189; 56,4%). O número absoluto de óbitos de agentes apresentou tendência decrescente ao longo da série (p = -0,78; IC95% -1,03 – -0,5). Entre as principais causas de morte estão acidentes de trânsito (n = 96; 28,7%), doenças cardiovasculares (n = 58; 17,3%), violência interpessoal (n = 51; 15,2%), suicídio (n = 35; 10,5%) e neoplasias malignas (n = 35; 10,4%). As mortes naturais predominaram entre os agentes com idade entre 51–73 anos (68,3%; IC95% 58,6–76,7) e mais de 26 anos de serviço (64,7%; IC95% 52,7–75,1), já as não naturais, entre a faixa etária de 19–35 anos (87,3%; IC95% 78,0–93,1) e de até 15 anos de serviço (70,2%; IC95% 63,1–76,4).

**CONCLUSÕES:**

Conclui-se que a tendência das mortes de agentes da polícia rodoviária federal foi decrescente no período. O conhecimento das causas de mortalidade pode auxiliar no desenvolvimento de políticas de prevenção de doenças e proteção à saúde desses policiais.

## INTRODUÇÃO

No desempenho de suas atividades ocupacionais, policiais executam uma ampla gama de tarefas arriscadas e potencialmente danosas à saúde^1 , 2^. Dentre os riscos inerentes dessa profissão estão incluídos esforços físicos intensos (perseguição a criminoso que resiste à prisão); eventos traumáticos (atendimento de acidentes com vítimas); estresse sonoro e químico (exposição a altos níveis de ruído e de dióxido de carbono); e ameaças à saúde física e psicológica (participação em confrontos armados)^1 , 3^. Devido às características do regime de trabalho dos agentes (trabalho por turnos), policiais podem sofrer alterações no ritmo circadiano e distúrbios do sono^4 , 5^.

Um estudo realizado com policiais de Nova York, observou um aumento significativo nas taxas de mortalidade para todas as causas de morte, neoplasias malignas, todas as neoplasias benignas, cirrose hepática e todas as doenças do sistema circulatório, entre os anos de 1950 e 2005^6^. No Brasil, estudo com policiais (civis e militares) e guardas municipais do estado do Rio de Janeiro indicou que agressões e acidentes de trânsito foram as principais causas de morte no período entre 1994 e 2004^7^. Ademais, esse grupo de trabalhadores também é acometido por outros problemas de saúde, como câncer e doenças cardiovasculares^2^, assim como apresentam maior exposição a situações de risco, inerentes ao exercício de sua profissão, o que pode gerar um aumento nas causas externas de morte, em decorrência de violência interpessoal e suicídio^7^.

Visto a presença de múltiplos fatores de risco à vitimização nessa profissão, tais acometimentos fornecem uma razão convincente para estudar as principais causas de mortalidade entre policiais. No entanto, até o momento, a população policial, em especial no Brasil, como os agentes da polícia rodoviária federal (PRF), vem sendo pouco estudada sob esse aspecto^6^. Diante disso, a geração de conhecimento científico sobre o mapeamento e categorização das principais causas de morte de agentes da PRF pode representar uma contribuição importante para os gestores no fomento de políticas públicas de saúde e melhores ações de capacitação profissional e projetos de prevenção de doenças e promoção à saúde dos servidores policiais.

Nesse sentido, o objetivo do presente estudo foi descrever e analisar a série temporal da mortalidade de agentes da PRF, em efetivo exercício, entre 1º de janeiro de 2001 e 31 de dezembro de 2020.

## MÉTODOS

Foi realizado um estudo observacional, com delineamento ecológico do tipo analítico-exploratório de séries temporais da mortalidade de agentes da PRF de todo o Brasil, no período de 2001 a 2020.

Foram incluídos no estudo os agentes em efetivo exercício da profissão, no período entre 1º de janeiro de 2001 e 31 de dezembro de 2020, que tiveram seus óbitos informados. Técnicos administrativos, assim como policiais que já estavam em regime de aposentadoria, foram excluídos do estudo.

Os dados sobre óbitos foram obtidos nos órgãos competentes, de acordo com a estrutura organizacional da PRF. A Figura 1 ilustra o processo amostral.

**Figura 1:** Fluxograma do processo amostral de obtenção dos dados sobre óbitos dentro da estrutura organizacional da PRF. PRF: polícia rodoviária federal; DICAD: Divisão de cadastro; APRF: Agentes da polícia federal.

As causas básicas de morte foram obtidas a partir do registro nas certidões de óbito de cada indivíduo, depois, foram agrupadas e codificadas pelos autores deste estudo, os quais são especialistas da área da saúde (EFM, FCF e JRVJ), conforme as categorias de causa de morte e suas definições em termos da classificação estatística internacional de doenças e problemas relacionados com a saúde em sua décima edição (CID-10)^12^. Foram classificadas como causas mal definidas/indeterminadas os casos em que a informação prestada na certidão de óbito constava como não determinada. As mortes foram categorizadas em naturais e não naturais, conforme classificação do Ministério da Saúde, utilizada em nossas análises.

As variáveis independentes incluídas foram o sexo (masculino e feminino); idade no momento do óbito (em anos completos); tempo de efetivo exercício no cargo (em anos completos); e região geográfica brasileira de lotação do servidor. As variáveis idade e tempo de exercício como agente foram categorizadas posteriormente, conforme melhor se distribuiu a população.

Os dados foram analisados com base em estatística descritiva pelo número de casos absolutos e relativos e seus respectivos intervalos de confiança de 95% (IC95%). Para análise da tendência de mortalidade, utilizou-se a regressão *Prais-Winsten* com variância robusta^13^. A variável dependente do modelo foi a frequência absoluta de mortes, a variável independente correspondeu aos anos da série histórica. A série foi considerada crescente, quando o coeficiente do modelo foi positivo, decrescente quando negativo e estacionário quando não houve diferença significante entre seu valor e o zero (p > 0,05)^13^. As análises bivariadas entre o desfecho e as exposições de interesse foram realizadas valendo-se do teste de qui-quadrado. Por fim, o desfecho também foi estudado a partir de sua taxa de incidência relativa, de acordo com o efetivo de policiais, em cada ano do período analisado. Para tanto, adotou-se o nível de significância de 5%. Todas as análises foram realizadas no pacote estatístico Stata 15.1.

É importante destacar que este estudo obteve aprovação do Comitê de Ética em Pesquisa com Seres Humanos da Escola Superior de Educação Física da Universidade Federal de Pelotas – RS, sob o número de protocolo 47809721.8.0000.5313.

## RESULTADOS

No período entre 2001 e 2020, foram registradas 346 mortes de agentes da PRF que estavam na ativa, sendo 11 mortes (3,2%) classificadas como indeterminadas, as quais foram excluídas das análises bivariadas, totalizando 335 óbitos com causas determinadas. Os agentes incluídos tinham em média 44,7 anos (DP = 10,8) e trabalhavam na PRF há 15,9 anos (DP = 10,6). Indivíduos do sexo masculino, com mais de 35 anos, com até 15 anos de serviço, e residentes na região Nordeste, estão entre os mais acometidos no período avaliado ( Tabela 1 ). Mortes provenientes de causas não naturais apresentaram maior proporção (56,4%) ( Tabela 1 ).

**Tabela 1:** Número e porcentagem de óbitos em agentes da polícia rodoviária federal, segundo sexo, idade, tempo de serviço, regiões e causa da morte, de acordo com a categorização do Ministério da Saúde. Brasil, 2001–2021 (n = 346).

	Óbitos
	
	n (%)
Sexo	
Masculino	333 (96,3)
Feminino	13 (3,7)
Idade (anos)	
19–35	81 (23,4)
36–50	160 (46,2)
51–73	105 (30,4)
Tempo de serviço (anos)	
≤ 15	185 (53,5)
16–25	90 (26,0)
≥ 26	71 (20,5)
Região	
Norte	35 (10,1)
Centro-Oeste	44 (12,7)
Nordeste	114 (33,0)
Sudeste	98 (28,3)
Sul	55 (15,9)
Causa da morte^a^	
Morte natural/doença	146 (43,6)
Morte não natural/causas externas	189 (56,4)

^a^11 informações de mortes foram perdidas (causa indeterminada).

A média de óbitos, ao longo do período, foi de 17,4 mortes/ano, com amplitude variando de sete a 29 mortes nos anos de 2013 e 2001, respectivamente, conforme demonstrado na Figura 2 . Analisando o período em décadas, é possível observar uma redução no número de mortes, tanto com relação à quantidade absoluta, quanto pelo valor médio, a partir da segunda década (2011–2020) em comparação ao período de 2001 até 2010 (12,3 e 22,3 mortes em média, respectivamente). Baseados nos resultados da regressão de *Prais-Winsten* , verifica-se que existe uma tendência significativa de diminuição do número de mortes ao longo do período, na ordem de -0,78 mortes por ano (IC95% -1,03 – -0,50; p < 0,001).

**Figura 2:** Frequências absoluta, relativa (%) e regressão de *Prais-Winsten* da estimativa de mortes ocorridas em agentes da polícia rodoviária federal nos últimos 20 anos, 2001–2020 (n = 346).

A Tabela 2 apresenta as causas específicas de mortes, em valores absolutos e relativos, segundo a classificação do Ministério da Saúde. A principal causa de morte observada são os acidentes de trânsito (28,7%), seguida pelas doenças cardiovasculares (17,3%), violência interpessoal (15,2%), suicídios (10,5%) e neoplasias malignas (10,4%). É possível observar que 54,4% do total de mortes no período estudado é atribuído a acidentes de trânsito, violência interpessoal e suicídio (mortes não naturais), bem como correspondem à 96,3% das mortes por causas não naturais/externas ao longo do período.

**Tabela 2:** Características das mortes em ordem decrescente entre 2001 e 2020 de agentes da polícia rodoviária federal em sua totalidade e estratificada conforme a categorização do Ministério da Saúde (n = 335).

Características	Mortes		

	Total	Naturais/doenças	Não naturais/causas externas
		
	n (%)	% (IC95%)	% (IC95%)
Acidente de trânsito	96 (28,7)	-	50,5 (43,4–57,6)
Doenças cardiovasculares	58 (17,3)	39,7 (32,1–47,9)	-
Violência interpessoal	51 (15,2)	-	26,8 (21,0–33,6)
Suicídio	36 (10,5)	-	18,9 (14,0–25,2)
Neoplasias malignas	35 (10,4)	24,0 (17,7–31,6)	-
Doenças digestivas	20 (6,0)	13,7 (9,0–20,3)	-
Doenças infecciosas e parasitárias	14 (4,2)	9,6 (5,8–15,6)	-
Infecção respiratória	12 (3,6)	8,2 (4,7–14,0)	-
Lesões não-intencionais	7 (2,1)	-	3,7 (1,8–7,6)
Doenças respiratórias	4 (1,2)	2,7 (1,0–7,1)	-
Doenças endócrinas	3 (0,9)	2,1 (0,7–6,2)	-

Na Tabela 3 , observa-se que a proporção de óbitos por causas não naturais é maior nos policiais com faixa etária entre 19 e 35 anos (87,3%; IC95% 78,0–93,1) e com tempo de serviço de até 15 anos (70,2%; IC95% 63,1–76,4). Já a proporção de óbitos por causas naturais é maior entre os agentes na faixa etária entre 51 e 73 anos (68,3%; IC95% 58,6–76,7) e com 26 ou mais anos de serviço (64,7%; IC95% 52,7–75,1%).

**Tabela 3:** Porcentagem de óbitos por categoria de morte em agentes da polícia rodoviária federal, segundo idade, tempo de serviço e região. Brasil, 2001–2021 (n = 335).

	Morte natural/doença	Morte não-natural/causas externas	p
		
	% (IC95%)	% (IC95%)	
Idade (anos)			< 0,001
19–35	12,7 (6,9–22,0)	87,3 (78,0–93,1)^a^	
36–50	43,0 (35,4–50,9)	57,0 (49,1–64,6)	
51–73	68,3 (58,6–76,7)^a^	31,7 (23,3–41,4)	
Tempo de serviço (anos)			< 0,001
≤ 15	29,8 (23,6–36,9)	70,2 (63,1–76,4)^a^	
16–25	55,8 (45,1–65,9)	44,2 (34,1–54,8)	
≥ 26	64,7 (52,7–75,1)^a^	35,3 (24,9–47,3)	
Região			0,64
Norte	38,7 (23,4–56,6)	61,3 (43,4–76,6)	
Centro-Oeste	36,4 (23,5–51,4)	63,6 (48,6–76,4)	
Nordeste	44,6 (35,7–53,9)	55,4 (46,0–64,3)	
Sudeste	48,9 (39,0–59,0)	51,1 (41,0–61,0)	
Sul	40,7 (28,5–54,2)	59,3 (45,8–71,5)	

IC95%: intervalo de confiança de 95%.

^a^Diferença estatística percentual de morte não natural/causas externas em relação a morte natural/doença.

A Figura 3 apresenta as taxas de mortalidade ocorridas no período analisado neste estudo, de 2001 a 2020. Dessa forma, sublinha-se que a taxa de mortalidade geral relativa a mil policiais vem decrescendo ao longo do período, sendo próxima a quatro mortes a cada mil policiais em 2001, chegando a taxas próximas a 1,5 mortes a cada mil policiais a partir de 2014. Além disso, é notável que a taxa de mortes não naturais foi maior em praticamente todos os anos analisados, com exceção dos anos de 2001, 2007, 2013 e 2019, em que a taxa de mortes naturais foi maior.

**Figura 3:** Taxa de incidência de mortalidade em agentes da polícia rodoviária federal entre 2001 e 2020, total e estratificada, conforme o critério do Ministério da Saúde.

## DISCUSSÃO

O presente estudo mostrou que a mortalidade por todas as causas é maior entre agentes do sexo masculino, com idade acima de 35 anos, 15 anos ou menos de trabalho e residentes na região Nordeste. Mortes por causas não naturais apresentaram maior prevalência, as quais são mais frequentes entre aqueles mais novos e com menor tempo de serviço. Uma tendência decrescente no número de mortes ao longo dos anos e uma queda na taxa de mortalidade ao longo do período foi observada. Acidentes de trânsito, doenças cardiovasculares, violência interpessoal, suicídio e neoplasias malignas estão entre as principais causas de mortes entre agentes da PRF.

Em relação ao perfil de idade e as causas de morte encontradas no período, observa-se que os policiais apresentam faixa etária e causas semelhantes às encontradas em outras populações policiais^11 , 14^. Merino^15^observou, a partir de um estudo ecológico de série temporal entre 2002 e 2006, que 77% dos policiais militares do estado de São Paulo morreram por causas não naturais. Assim, percebe-se que esse grupo populacional está exposto a fatores de risco específicos da profissão (confrontos armados, perseguições a veículos) o que pode estar relacionado a essa maior proporção de mortes não naturais.

Além disso, o maior percentual de óbitos por causas não naturais entre os agentes com menor tempo de serviço corrobora o achado dentre policiais militares do Rio de Janeiro, onde aqueles com menor tempo de serviço apresentaram risco aumentado em 2,4 vezes de morrer, quando comparado aos com maior tempo^16^. Falta de percepção e vivência dos riscos das atividades da profissão pelos policiais mais novos pode explicar essa relação. Em policiais novatos a percepção de adrenalina produzida pelo inusitado e sua motivação para ação na profissão diminui a percepção do risco nos momentos de perseguições ou confrontos, colocando esses indivíduos em risco aumentado^17^.

Diante disso, os agentes da PRF estão, como outras forças policiais, mais vulneráveis às mortes não naturais (violência interpessoal, acidentes de trânsito e suicídio), visto que entre suas competências estão, no âmbito das rodovias federais, o patrulhamento ostensivo e a repressão a todos os tipos de crimes^18^, o que os expõem a um maior risco de sua integridade física. Assim, o conhecimento desse perfil de vitimização dos policiais possibilita uma identificação do padrão de monitoramento mais fidedigno e pode auxiliar na avaliação de prioridades dos investimentos em políticas de saúde para agentes da PRF, visando a implementação de ações/programas mais efetivos na redução da mortalidade, que podem promover a diminuição dos custos para a administração pública em geral.

Entretanto, a PRF não possui em seu quadro oficial de servidores cargos relacionados a profissionais da área de saúde nem setores de saúde como ambulatórios, policlínicas e hospitais. No entanto, em sua estrutura organizacional, há setores de gestão de saúde, que atuam em nível básico de atendimento, com intervenções genéricas (aplicação de testes físicos^19^, palestras com orientações de estilos de vida saudáveis^20^), processuais (registro de atestados médicos e encaminhamentos para perícias oficiais), e de gestão de crises/incidentes críticos (teleatendimento do servidor envolvido em confronto armado, acidente de trânsito e adoecido por covid-19).

Uma redução no número absoluto de mortes foi observada, na última década da série temporal (2011–2020), o que pode estar relacionada à implementação de programas e ações de prevenção e promoção da saúde na PRF, como: i) Patrulha da saúde, cuja finalidade é promover conhecimento sobre medidas de prevenção a doenças e promoção de um estilo de vida saudável, conforme dados previamente publicados^20^; ii) Disciplina relacionada à saúde, na matriz curricular dos cursos de formação policial, a qual tem por objetivo a adoção de hábitos que promovam a saúde integral dos agentes em formação, com foco em técnicas e cuidados que envolvam a prática de exercícios físicos regulares, alimentação saudável, qualidade do sono e questões relativas à saúde psicoemocional; iii) Teste de aptidão física, o qual visa aferir os níveis de aptidão física dos agentes para fins de promoção na carreira e critério de seleção para atividades específicas^21^; e iv) Programa de educação física institucional, o qual incentiva à prática de atividade física fora do ambiente de trabalho^19^. Tais ações podem ter contribuído para mudanças de estilo de vida e, consequentemente, redução da morbimortalidade por causas naturais entre os agentes, porém, pelo delineamento do estudo realizado, não é possível analisar a causalidade entre as iniciativas de saúde na PRF e a mortalidade ao longo do período^13^.

Os acidentes de trânsito foram considerados como a principal causa de morte entre os agentes durante o período de 2001 a 2020 (28,7%), corroborando estudo recente que descreveu a mortalidade entre policiais militares do Rio Grande do Sul entre os anos de 2006 e 2016^14^. Limeira e Donato^14^observaram que o acidente de trânsito foi a principal causa de morte entre policiais da ativa em serviço, com 41,3% do total de mortes no período estudado, estando a frente das mortes por violência interpessoal (homicídio) e suicídio, conforme ocorreu entre os agentes deste estudo durante os 20 anos. Tanto agentes da PRF, como policiais militares, fazem parte da polícia ostensiva da União e dos estados, respectivamente, cujas atividades de policiamento exigem o constante uso de carros e motos (viaturas), assim como, para manutenção da segurança viária, permanecem em locais de grande volume de veículos, o que pode aumentar o risco de atropelamentos. Nesse contexto, é relevante a institucionalização de políticas de prevenção, a partir de capacitações dos agentes e pesquisas sobre o assunto, visando melhorar suas condutas em ocorrências de acidentes de trânsito.

As doenças cardiovasculares foram a principal causa de morte entre as mortes naturais ao longo da série temporal estudada (39,7%), sendo semelhante ao encontrado em estudos com policiais novaiorquinos^6^e paulistanos^15^. Vena et al.^6^relataram um elevado número de mortes por doenças relacionadas ao sistema circulatório (44,9% de todas as mortes) em policiais de Nova York entre 1950 e 2005. Esse elevado percentual pode ser justificado pela prevalência elevada de alguns fatores de risco cardiovasculares presentes na população policial, como turnos longos de trabalho, distúrbios de sono, estresse psicológico, estresse físico imprevisto e exposição a ruído^2^.

Além disso, os resultados de Vena et al.^9^apontam que policiais americanos tiveram um aumento nas taxas de mortes por doenças cardíacas arterioscleróticas e por diferentes tipos de cânceres entre 1950 e 1970. Similarmente, Forastiere et al.^8^estudando uma coorte histórica de policiais em Roma, na Itália, observaram aumento nas taxas de mortalidade por doença isquêmica do coração em policiais com menos de 50 anos de idade, bem como para alguns tipos de cânceres. Violanti et al.^10^, ao examinarem uma coorte de policiais americanos entre os anos de 1950 e 1990, encontraram um significativo aumento na mortalidade por todas as neoplasias malignas combinadas, bem como por doença cardíaca arteriosclerótica em policiais com maior tempo de serviço. Em resumo, esses resultados, bem como os do presente estudo, reforçam a relação entre tempo de serviço e idade na mortalidade policial por causas naturais, especialmente por doenças cardiovasculares e neoplasias malignas, possivelmente relacionadas aos fatores de risco ocupacionais e de estilo de vida dos policiais^2^.

Outro ponto importante é que, durante os 20 anos estudados, os suicídios de agentes da PRF estão entre as quatro principais causas de óbitos, sendo a terceira por causas externas, o que corrobora os achados prévios de estudos com policiais militares^14 , 15^. A profissão policial expõe seus profissionais a características (fácil acesso a arma de fogo, por exemplo) e situações específicas da profissão (confrontos armados, violência e convivência com cenas de óbitos, etc.), as quais podem ser determinantes para o aumento do risco de suicídio nessa população^22^. Dessa forma, qualquer instituição policial deve monitorar e analisar os dados sobre o perfil e a taxa de óbitos por suicídio, a fim de orientar a formulação, implementação e avaliação de políticas públicas na área de saúde mental policial.

Tendo por base a análise de tendência do número de mortes absolutas e a taxa de incidência de mortalidade, encontradas neste estudo, a redução demonstrada na série está em discordância com os resultados apresentados em estudo com policiais militares do Rio de Janeiro^16^. Minayo et al.^16^reuniram dados de taxa de mortalidade nessa população, os quais apresentaram tendência crescente no período entre 1994 e 2004, com valores entre 12,1 e 17 mortes por mil policiais entre os anos de 2000 e 2004. Esses valores estão muito acima dos encontrados, nos respectivos anos neste estudo, o que pode ser justificado pelas características distintas entre o perfil dos policiais de cada instituição (PRF e policiais militares do Rio de Janeiro), visto que o percentual de policiais militares que trabalham em outra atividade laboral na folga (principalmente em serviços de segurança privada) é muito elevado^16^.

Reconhece-se que este estudo tem limitações. Primeiramente, a qualidade de dados secundários é dependente do sistema de informação de onde os dados são originados. No caso deste estudo, as informações de óbito foram retiradas das certidões de óbitos, as quais são preenchidas nos cartórios a partir da declaração de óbito, que é preenchida por um médico. Assim, tanto no preenchimento das certidões de óbitos quanto nas transcrições da declaração de óbito há limitações de padronização que dificultaram a classificação das causas de morte^25^. Em segundo lugar, é importante destacar a ausência de dados completos da população, como presença de doenças preexistentes, medidas de aptidão física, atividade policial ou militar prévia ao ingresso na PRF, dentre outras exposições que poderiam auxiliar em uma análise de possíveis fatores de risco para mortalidade. Entretanto, o presente estudo destaca-se por seu caráter de ineditismo sobre o tema da mortalidade policial em âmbito nacional, bem como a amplitude (20 anos) e o período atual de avaliação da série temporal (2001–2020).

De acordo com os dados apresentados neste estudo será possível traçar o diagnóstico situacional das mortes de agentes da PRF no Brasil. Tais informações serão importantes para subsidiar a formulação de políticas públicas de saúde, bem como de ações práticas de capacitação profissional na PRF baseadas em evidências.

Os resultados deste artigo sugerem que as políticas públicas de saúde empregadas pela PRF podem ter contribuído para uma redução no número de mortes de agentes em efetivo exercício por todas as causas ao longo do período de 2001 a 2020. Contudo, devem ser implementadas ações e programas relacionados à prevenção das principais causas de morte entre os agentes, principalmente, por acidentes de trânsito, doenças cardiovasculares, violência interpessoal, suicídio e neoplasias malignas. Essas medidas precisam ser implementadas visando atingir o maior número de agentes, a fim de reduzir os impactos, tanto na saúde como nos custos decorrentes dela.
